# Supplementary material for: Multi‐dataset identification of innovative feature genes and molecular mechanisms in keratoconus
Source: J Cell Mol Med. 2024 Sep 19;28(18):e70079. doi: 10.1111/jcmm.70079 (PMC11412914; doi:10.1111/jcmm.70079)
Supplement: Supplementary file 2 — Material S2. [file JCMM-28-e70079-s002.zip › 7.GSEA analysis/5.RNF217-AS1/gsea_report_for_l_1678852909625.html]

Report for l 1678852909625 [GSEA]

| GS  follow link to MSigDB | GS DETAILS | SIZE | ES | NES | NOM p-val | FDR q-val | FWER p-val | RANK AT MAX | LEADING EDGE || 1 | KEGG\_COLORECTAL\_CANCER | Details ... | 62 | -0.44 | -1.74 | 0.012 | 0.567 | 0.292 | 4395 | tags=18%, list=13%, signal=20% |
| 2 | KEGG\_ENDOMETRIAL\_CANCER | Details ... | 52 | -0.45 | -1.72 | 0.010 | 0.371 | 0.349 | 4395 | tags=17%, list=13%, signal=20% |
| 3 | KEGG\_BASAL\_CELL\_CARCINOMA | Details ... | 55 | -0.56 | -1.69 | 0.010 | 0.352 | 0.429 | 4650 | tags=31%, list=13%, signal=36% |
| 4 | KEGG\_GLYCOSPHINGOLIPID\_BIOSYNTHESIS\_LACTO\_AND\_NEOLACTO\_SERIES | Details ... | 26 | -0.56 | -1.64 | 0.014 | 0.397 | 0.527 | 4614 | tags=27%, list=13%, signal=31% |
| 5 | KEGG\_MELANOGENESIS | Details ... | 101 | -0.43 | -1.64 | 0.006 | 0.327 | 0.532 | 3758 | tags=20%, list=11%, signal=22% |
| 6 | KEGG\_GLYCOSAMINOGLYCAN\_BIOSYNTHESIS\_CHONDROITIN\_SULFATE | Details ... | 22 | -0.61 | -1.63 | 0.020 | 0.301 | 0.550 | 9099 | tags=55%, list=26%, signal=74% |
| 7 | KEGG\_BASE\_EXCISION\_REPAIR | Details ... | 33 | -0.51 | -1.59 | 0.030 | 0.355 | 0.609 | 13487 | tags=64%, list=39%, signal=104% |
| 8 | KEGG\_OXIDATIVE\_PHOSPHORYLATION | Details ... | 98 | -0.37 | -1.59 | 0.046 | 0.320 | 0.615 | 5544 | tags=10%, list=16%, signal=12% |
| 9 | KEGG\_CELL\_CYCLE | Details ... | 124 | -0.43 | -1.57 | 0.090 | 0.340 | 0.659 | 10947 | tags=40%, list=32%, signal=59% |
| 10 | KEGG\_FC\_GAMMA\_R\_MEDIATED\_PHAGOCYTOSIS | Details ... | 92 | -0.40 | -1.56 | 0.024 | 0.331 | 0.674 | 3200 | tags=13%, list=9%, signal=14% |
| 11 | KEGG\_INOSITOL\_PHOSPHATE\_METABOLISM | Details ... | 54 | -0.40 | -1.54 | 0.054 | 0.360 | 0.712 | 9138 | tags=30%, list=26%, signal=40% |
| 12 | KEGG\_DNA\_REPLICATION | Details ... | 36 | -0.45 | -1.53 | 0.038 | 0.356 | 0.725 | 13010 | tags=75%, list=37%, signal=120% |
| 13 | KEGG\_WNT\_SIGNALING\_PATHWAY | Details ... | 149 | -0.36 | -1.52 | 0.012 | 0.354 | 0.741 | 5443 | tags=21%, list=16%, signal=25% |
| 14 | KEGG\_CELL\_ADHESION\_MOLECULES\_CAMS | Details ... | 130 | -0.47 | -1.51 | 0.010 | 0.334 | 0.744 | 5673 | tags=30%, list=16%, signal=36% |
| 15 | KEGG\_LYSINE\_DEGRADATION | Details ... | 39 | -0.47 | -1.51 | 0.057 | 0.319 | 0.746 | 9471 | tags=44%, list=27%, signal=60% |
| 16 | KEGG\_LYSOSOME | Details ... | 117 | -0.29 | -1.51 | 0.093 | 0.305 | 0.751 | 14374 | tags=50%, list=41%, signal=84% |
| 17 | KEGG\_AMINOACYL\_TRNA\_BIOSYNTHESIS | Details ... | 22 | -0.49 | -1.49 | 0.042 | 0.321 | 0.772 | 13358 | tags=68%, list=38%, signal=111% |
| 18 | KEGG\_O\_GLYCAN\_BIOSYNTHESIS | Details ... | 29 | -0.53 | -1.48 | 0.061 | 0.322 | 0.781 | 4111 | tags=28%, list=12%, signal=31% |
| 19 | KEGG\_CHEMOKINE\_SIGNALING\_PATHWAY | Details ... | 183 | -0.42 | -1.48 | 0.034 | 0.316 | 0.790 | 4933 | tags=23%, list=14%, signal=27% |
| 20 | KEGG\_SELENOAMINO\_ACID\_METABOLISM | Details ... | 24 | -0.50 | -1.47 | 0.068 | 0.310 | 0.800 | 10965 | tags=42%, list=32%, signal=61% |
| 21 | KEGG\_NUCLEOTIDE\_EXCISION\_REPAIR | Details ... | 43 | -0.34 | -1.47 | 0.018 | 0.302 | 0.803 | 12423 | tags=53%, list=36%, signal=83% |
| 22 | KEGG\_ONE\_CARBON\_POOL\_BY\_FOLATE | Details ... | 17 | -0.53 | -1.47 | 0.068 | 0.291 | 0.805 | 11283 | tags=59%, list=32%, signal=87% |
| 23 | KEGG\_PROSTATE\_CANCER | Details ... | 89 | -0.34 | -1.46 | 0.036 | 0.288 | 0.808 | 3428 | tags=12%, list=10%, signal=14% |
| 24 | KEGG\_PYRIMIDINE\_METABOLISM | Details ... | 95 | -0.33 | -1.45 | 0.130 | 0.302 | 0.825 | 13000 | tags=47%, list=37%, signal=76% |
| 25 | KEGG\_GLYCOSYLPHOSPHATIDYLINOSITOL\_GPI\_ANCHOR\_BIOSYNTHESIS | Details ... | 25 | -0.46 | -1.43 | 0.143 | 0.334 | 0.845 | 11271 | tags=64%, list=32%, signal=95% |
| 26 | KEGG\_ACUTE\_MYELOID\_LEUKEMIA | Details ... | 57 | -0.34 | -1.41 | 0.083 | 0.352 | 0.852 | 4395 | tags=14%, list=13%, signal=16% |
| 27 | KEGG\_THYROID\_CANCER | Details ... | 29 | -0.41 | -1.40 | 0.073 | 0.359 | 0.864 | 3946 | tags=14%, list=11%, signal=16% |
| 28 | KEGG\_N\_GLYCAN\_BIOSYNTHESIS | Details ... | 46 | -0.42 | -1.39 | 0.193 | 0.380 | 0.884 | 12498 | tags=46%, list=36%, signal=71% |
| 29 | KEGG\_GLUTATHIONE\_METABOLISM | Details ... | 49 | -0.42 | -1.39 | 0.128 | 0.370 | 0.884 | 9805 | tags=41%, list=28%, signal=57% |
| 30 | KEGG\_FC\_EPSILON\_RI\_SIGNALING\_PATHWAY | Details ... | 78 | -0.39 | -1.38 | 0.062 | 0.375 | 0.888 | 4395 | tags=17%, list=13%, signal=19% |
| 31 | KEGG\_BASAL\_TRANSCRIPTION\_FACTORS | Details ... | 33 | -0.32 | -1.38 | 0.149 | 0.365 | 0.889 | 13763 | tags=55%, list=40%, signal=90% |
| 32 | KEGG\_HUNTINGTONS\_DISEASE | Details ... | 158 | -0.24 | -1.38 | 0.110 | 0.356 | 0.891 | 16211 | tags=59%, list=47%, signal=110% |
| 33 | KEGG\_BUTANOATE\_METABOLISM | Details ... | 34 | -0.47 | -1.37 | 0.130 | 0.355 | 0.896 | 3322 | tags=21%, list=10%, signal=23% |
| 34 | KEGG\_VASOPRESSIN\_REGULATED\_WATER\_REABSORPTION | Details ... | 44 | -0.39 | -1.37 | 0.109 | 0.352 | 0.899 | 14296 | tags=55%, list=41%, signal=93% |
| 35 | KEGG\_PANTOTHENATE\_AND\_COA\_BIOSYNTHESIS | Details ... | 16 | -0.53 | -1.35 | 0.135 | 0.383 | 0.917 | 2352 | tags=25%, list=7%, signal=27% |
| 36 | KEGG\_COMPLEMENT\_AND\_COAGULATION\_CASCADES | Details ... | 69 | -0.42 | -1.34 | 0.061 | 0.378 | 0.923 | 2930 | tags=20%, list=8%, signal=22% |
| 37 | KEGG\_PHOSPHATIDYLINOSITOL\_SIGNALING\_SYSTEM | Details ... | 76 | -0.32 | -1.34 | 0.133 | 0.372 | 0.924 | 4888 | tags=16%, list=14%, signal=18% |
| 38 | KEGG\_EPITHELIAL\_CELL\_SIGNALING\_IN\_HELICOBACTER\_PYLORI\_INFECTION | Details ... | 66 | -0.39 | -1.34 | 0.168 | 0.363 | 0.924 | 4520 | tags=17%, list=13%, signal=19% |
| 39 | KEGG\_PYRUVATE\_METABOLISM | Details ... | 40 | -0.34 | -1.33 | 0.162 | 0.370 | 0.931 | 14153 | tags=55%, list=41%, signal=93% |
| 40 | KEGG\_PARKINSONS\_DISEASE | Details ... | 98 | -0.32 | -1.33 | 0.180 | 0.361 | 0.931 | 16211 | tags=60%, list=47%, signal=113% |
| 41 | KEGG\_P53\_SIGNALING\_PATHWAY | Details ... | 66 | -0.35 | -1.31 | 0.188 | 0.400 | 0.946 | 8334 | tags=29%, list=24%, signal=38% |
| 42 | KEGG\_VIBRIO\_CHOLERAE\_INFECTION | Details ... | 51 | -0.31 | -1.31 | 0.137 | 0.398 | 0.947 | 4520 | tags=16%, list=13%, signal=18% |
| 43 | KEGG\_ASTHMA | Details ... | 29 | -0.58 | -1.30 | 0.173 | 0.403 | 0.954 | 4620 | tags=45%, list=13%, signal=52% |
| 44 | KEGG\_TGF\_BETA\_SIGNALING\_PATHWAY | Details ... | 85 | -0.39 | -1.29 | 0.151 | 0.405 | 0.958 | 5875 | tags=26%, list=17%, signal=31% |
| 45 | KEGG\_UBIQUITIN\_MEDIATED\_PROTEOLYSIS | Details ... | 130 | -0.22 | -1.29 | 0.173 | 0.401 | 0.961 | 11408 | tags=29%, list=33%, signal=43% |
| 46 | KEGG\_MELANOMA | Details ... | 71 | -0.38 | -1.29 | 0.120 | 0.397 | 0.962 | 4395 | tags=18%, list=13%, signal=21% |
| 47 | KEGG\_TYROSINE\_METABOLISM | Details ... | 40 | -0.40 | -1.27 | 0.111 | 0.419 | 0.966 | 2002 | tags=15%, list=6%, signal=16% |
| 48 | KEGG\_SYSTEMIC\_LUPUS\_ERYTHEMATOSUS | Details ... | 56 | -0.52 | -1.26 | 0.218 | 0.440 | 0.968 | 4620 | tags=30%, list=13%, signal=35% |
| 49 | KEGG\_PROPANOATE\_METABOLISM | Details ... | 31 | -0.39 | -1.26 | 0.242 | 0.433 | 0.968 | 12154 | tags=52%, list=35%, signal=79% |
| 50 | KEGG\_PRIMARY\_BILE\_ACID\_BIOSYNTHESIS | Details ... | 16 | -0.55 | -1.25 | 0.196 | 0.431 | 0.969 | 6370 | tags=50%, list=18%, signal=61% |
| 51 | KEGG\_PATHWAYS\_IN\_CANCER |  | 322 | -0.29 | -1.25 | 0.177 | 0.433 | 0.973 | 4788 | tags=14%, list=14%, signal=16% |
| 52 | KEGG\_NON\_SMALL\_CELL\_LUNG\_CANCER |  | 54 | -0.27 | -1.24 | 0.210 | 0.433 | 0.974 | 4395 | tags=13%, list=13%, signal=15% |
| 53 | KEGG\_PURINE\_METABOLISM |  | 151 | -0.29 | -1.23 | 0.223 | 0.451 | 0.978 | 6166 | tags=19%, list=18%, signal=22% |
| 54 | KEGG\_LEISHMANIA\_INFECTION |  | 71 | -0.44 | -1.23 | 0.228 | 0.446 | 0.978 | 3861 | tags=21%, list=11%, signal=24% |
| 55 | KEGG\_NICOTINATE\_AND\_NICOTINAMIDE\_METABOLISM |  | 24 | -0.46 | -1.23 | 0.247 | 0.444 | 0.978 | 6759 | tags=38%, list=19%, signal=47% |
| 56 | KEGG\_PROXIMAL\_TUBULE\_BICARBONATE\_RECLAMATION |  | 23 | -0.40 | -1.22 | 0.176 | 0.439 | 0.978 | 7541 | tags=48%, list=22%, signal=61% |
| 57 | KEGG\_BETA\_ALANINE\_METABOLISM |  | 22 | -0.43 | -1.21 | 0.204 | 0.462 | 0.982 | 7394 | tags=36%, list=21%, signal=46% |
| 58 | KEGG\_INTESTINAL\_IMMUNE\_NETWORK\_FOR\_IGA\_PRODUCTION |  | 48 | -0.45 | -1.21 | 0.217 | 0.459 | 0.982 | 6722 | tags=42%, list=19%, signal=52% |
| 59 | KEGG\_MISMATCH\_REPAIR |  | 23 | -0.34 | -1.21 | 0.210 | 0.452 | 0.982 | 12976 | tags=74%, list=37%, signal=118% |
| 60 | KEGG\_HEDGEHOG\_SIGNALING\_PATHWAY |  | 55 | -0.37 | -1.20 | 0.186 | 0.457 | 0.984 | 5443 | tags=24%, list=16%, signal=28% |
| 61 | KEGG\_REGULATION\_OF\_ACTIN\_CYTOSKELETON |  | 210 | -0.28 | -1.20 | 0.195 | 0.456 | 0.986 | 4654 | tags=12%, list=13%, signal=14% |
| 62 | KEGG\_VIRAL\_MYOCARDITIS |  | 70 | -0.38 | -1.19 | 0.254 | 0.466 | 0.990 | 8033 | tags=36%, list=23%, signal=46% |
| 63 | KEGG\_ENDOCYTOSIS |  | 179 | -0.22 | -1.18 | 0.263 | 0.466 | 0.990 | 7300 | tags=15%, list=21%, signal=18% |
| 64 | KEGG\_ETHER\_LIPID\_METABOLISM |  | 29 | -0.46 | -1.18 | 0.240 | 0.460 | 0.990 | 5587 | tags=24%, list=16%, signal=29% |
| 65 | KEGG\_RNA\_POLYMERASE |  | 27 | -0.37 | -1.18 | 0.259 | 0.461 | 0.990 | 15633 | tags=59%, list=45%, signal=108% |
| 66 | KEGG\_MAPK\_SIGNALING\_PATHWAY |  | 265 | -0.28 | -1.18 | 0.210 | 0.455 | 0.990 | 4065 | tags=14%, list=12%, signal=16% |
| 67 | KEGG\_PRION\_DISEASES |  | 35 | -0.41 | -1.17 | 0.252 | 0.466 | 0.992 | 3368 | tags=20%, list=10%, signal=22% |
| 68 | KEGG\_AXON\_GUIDANCE |  | 128 | -0.30 | -1.15 | 0.270 | 0.497 | 0.994 | 6383 | tags=22%, list=18%, signal=27% |
| 69 | KEGG\_VALINE\_LEUCINE\_AND\_ISOLEUCINE\_DEGRADATION |  | 43 | -0.32 | -1.14 | 0.293 | 0.497 | 0.995 | 12377 | tags=53%, list=36%, signal=83% |
| 70 | KEGG\_CARDIAC\_MUSCLE\_CONTRACTION |  | 72 | -0.30 | -1.13 | 0.218 | 0.519 | 0.997 | 4302 | tags=17%, list=12%, signal=19% |
| 71 | KEGG\_SPHINGOLIPID\_METABOLISM |  | 35 | -0.33 | -1.13 | 0.322 | 0.522 | 0.998 | 6074 | tags=23%, list=17%, signal=28% |
| 72 | KEGG\_GLYCOSAMINOGLYCAN\_BIOSYNTHESIS\_HEPARAN\_SULFATE |  | 26 | -0.33 | -1.10 | 0.321 | 0.559 | 0.998 | 9099 | tags=42%, list=26%, signal=57% |
| 73 | KEGG\_TOLL\_LIKE\_RECEPTOR\_SIGNALING\_PATHWAY |  | 96 | -0.31 | -1.09 | 0.343 | 0.572 | 0.998 | 4904 | tags=19%, list=14%, signal=22% |
| 74 | KEGG\_OTHER\_GLYCAN\_DEGRADATION |  | 15 | -0.38 | -1.08 | 0.370 | 0.591 | 0.999 | 11850 | tags=47%, list=34%, signal=71% |
| 75 | KEGG\_ADIPOCYTOKINE\_SIGNALING\_PATHWAY |  | 66 | -0.30 | -1.08 | 0.389 | 0.584 | 0.999 | 8717 | tags=30%, list=25%, signal=40% |
| 76 | KEGG\_GLYCOLYSIS\_GLUCONEOGENESIS |  | 61 | -0.30 | -1.08 | 0.361 | 0.589 | 0.999 | 12133 | tags=48%, list=35%, signal=73% |
| 77 | KEGG\_ARRHYTHMOGENIC\_RIGHT\_VENTRICULAR\_CARDIOMYOPATHY\_ARVC |  | 74 | -0.35 | -1.08 | 0.355 | 0.581 | 0.999 | 7146 | tags=27%, list=21%, signal=34% |
| 78 | KEGG\_HISTIDINE\_METABOLISM |  | 28 | -0.40 | -1.07 | 0.396 | 0.583 | 0.999 | 11606 | tags=57%, list=33%, signal=86% |
| 79 | KEGG\_PEROXISOME |  | 78 | -0.27 | -1.07 | 0.402 | 0.583 | 0.999 | 5583 | tags=17%, list=16%, signal=20% |
| 80 | KEGG\_ANTIGEN\_PROCESSING\_AND\_PRESENTATION |  | 79 | -0.33 | -1.06 | 0.383 | 0.588 | 0.999 | 7938 | tags=30%, list=23%, signal=39% |
| 81 | KEGG\_LEUKOCYTE\_TRANSENDOTHELIAL\_MIGRATION |  | 114 | -0.28 | -1.05 | 0.349 | 0.602 | 0.999 | 2507 | tags=11%, list=7%, signal=12% |
| 82 | KEGG\_FRUCTOSE\_AND\_MANNOSE\_METABOLISM |  | 32 | -0.38 | -1.05 | 0.410 | 0.598 | 0.999 | 7555 | tags=25%, list=22%, signal=32% |
| 83 | KEGG\_GALACTOSE\_METABOLISM |  | 25 | -0.35 | -1.04 | 0.424 | 0.604 | 0.999 | 12938 | tags=48%, list=37%, signal=76% |
| 84 | KEGG\_CYTOSOLIC\_DNA\_SENSING\_PATHWAY |  | 47 | -0.31 | -1.04 | 0.394 | 0.603 | 0.999 | 4904 | tags=17%, list=14%, signal=20% |
| 85 | KEGG\_NITROGEN\_METABOLISM |  | 23 | -0.41 | -1.03 | 0.407 | 0.620 | 0.999 | 7919 | tags=48%, list=23%, signal=62% |
| 86 | KEGG\_HYPERTROPHIC\_CARDIOMYOPATHY\_HCM |  | 83 | -0.30 | -1.02 | 0.420 | 0.629 | 0.999 | 5865 | tags=24%, list=17%, signal=29% |
| 87 | KEGG\_ALANINE\_ASPARTATE\_AND\_GLUTAMATE\_METABOLISM |  | 30 | -0.31 | -1.01 | 0.421 | 0.638 | 1.000 | 5515 | tags=23%, list=16%, signal=28% |
| 88 | KEGG\_PANCREATIC\_CANCER |  | 69 | -0.24 | -1.01 | 0.426 | 0.638 | 1.000 | 4395 | tags=12%, list=13%, signal=13% |
| 89 | KEGG\_CHRONIC\_MYELOID\_LEUKEMIA |  | 73 | -0.26 | -1.01 | 0.419 | 0.636 | 1.000 | 11836 | tags=34%, list=34%, signal=52% |
| 90 | KEGG\_TIGHT\_JUNCTION |  | 127 | -0.27 | -1.01 | 0.427 | 0.633 | 1.000 | 7833 | tags=25%, list=23%, signal=32% |
| 91 | KEGG\_GLYCOSAMINOGLYCAN\_BIOSYNTHESIS\_KERATAN\_SULFATE |  | 15 | -0.34 | -0.95 | 0.503 | 0.740 | 1.000 | 4614 | tags=27%, list=13%, signal=31% |
| 92 | KEGG\_ARGININE\_AND\_PROLINE\_METABOLISM |  | 52 | -0.27 | -0.95 | 0.524 | 0.732 | 1.000 | 9994 | tags=37%, list=29%, signal=51% |
| 93 | KEGG\_HEMATOPOIETIC\_CELL\_LINEAGE |  | 87 | -0.31 | -0.95 | 0.520 | 0.727 | 1.000 | 8478 | tags=41%, list=24%, signal=55% |
| 94 | KEGG\_DORSO\_VENTRAL\_AXIS\_FORMATION |  | 24 | -0.29 | -0.95 | 0.516 | 0.734 | 1.000 | 4345 | tags=13%, list=13%, signal=14% |
| 95 | KEGG\_DILATED\_CARDIOMYOPATHY |  | 90 | -0.26 | -0.94 | 0.552 | 0.736 | 1.000 | 4584 | tags=19%, list=13%, signal=22% |
| 96 | KEGG\_LONG\_TERM\_DEPRESSION |  | 67 | -0.28 | -0.94 | 0.517 | 0.729 | 1.000 | 6562 | tags=22%, list=19%, signal=28% |
| 97 | KEGG\_GRAFT\_VERSUS\_HOST\_DISEASE |  | 39 | -0.37 | -0.93 | 0.536 | 0.740 | 1.000 | 4619 | tags=26%, list=13%, signal=30% |
| 98 | KEGG\_INSULIN\_SIGNALING\_PATHWAY |  | 136 | -0.21 | -0.93 | 0.537 | 0.740 | 1.000 | 8528 | tags=21%, list=25%, signal=28% |
| 99 | KEGG\_APOPTOSIS |  | 87 | -0.23 | -0.93 | 0.557 | 0.737 | 1.000 | 3368 | tags=11%, list=10%, signal=13% |
| 100 | KEGG\_PRIMARY\_IMMUNODEFICIENCY |  | 35 | -0.34 | -0.93 | 0.562 | 0.737 | 1.000 | 6618 | tags=26%, list=19%, signal=32% |
| 101 | KEGG\_ALDOSTERONE\_REGULATED\_SODIUM\_REABSORPTION |  | 42 | -0.27 | -0.92 | 0.549 | 0.733 | 1.000 | 5154 | tags=21%, list=15%, signal=25% |
| 102 | KEGG\_SMALL\_CELL\_LUNG\_CANCER |  | 84 | -0.24 | -0.92 | 0.531 | 0.734 | 1.000 | 4788 | tags=14%, list=14%, signal=17% |
| 103 | KEGG\_HOMOLOGOUS\_RECOMBINATION |  | 26 | -0.28 | -0.92 | 0.531 | 0.729 | 1.000 | 13112 | tags=58%, list=38%, signal=93% |
| 104 | KEGG\_ALPHA\_LINOLENIC\_ACID\_METABOLISM |  | 18 | -0.39 | -0.92 | 0.553 | 0.725 | 1.000 | 5587 | tags=22%, list=16%, signal=26% |
| 105 | KEGG\_PROGESTERONE\_MEDIATED\_OOCYTE\_MATURATION |  | 85 | -0.22 | -0.92 | 0.548 | 0.722 | 1.000 | 3368 | tags=9%, list=10%, signal=10% |
| 106 | KEGG\_ALLOGRAFT\_REJECTION |  | 37 | -0.38 | -0.91 | 0.591 | 0.721 | 1.000 | 4620 | tags=27%, list=13%, signal=31% |
| 107 | KEGG\_ARACHIDONIC\_ACID\_METABOLISM |  | 57 | -0.27 | -0.91 | 0.651 | 0.722 | 1.000 | 5587 | tags=19%, list=16%, signal=23% |
| 108 | KEGG\_CYTOKINE\_CYTOKINE\_RECEPTOR\_INTERACTION |  | 256 | -0.30 | -0.91 | 0.604 | 0.716 | 1.000 | 7247 | tags=30%, list=21%, signal=38% |
| 109 | KEGG\_ALZHEIMERS\_DISEASE |  | 144 | -0.17 | -0.90 | 0.594 | 0.718 | 1.000 | 2359 | tags=3%, list=7%, signal=4% |
| 110 | KEGG\_LINOLEIC\_ACID\_METABOLISM |  | 28 | -0.34 | -0.90 | 0.617 | 0.718 | 1.000 | 1717 | tags=14%, list=5%, signal=15% |
| 111 | KEGG\_STEROID\_HORMONE\_BIOSYNTHESIS |  | 46 | -0.29 | -0.90 | 0.676 | 0.724 | 1.000 | 7974 | tags=39%, list=23%, signal=51% |
| 112 | KEGG\_CALCIUM\_SIGNALING\_PATHWAY |  | 177 | -0.23 | -0.89 | 0.719 | 0.729 | 1.000 | 6549 | tags=23%, list=19%, signal=28% |
| 113 | KEGG\_NOD\_LIKE\_RECEPTOR\_SIGNALING\_PATHWAY |  | 61 | -0.28 | -0.89 | 0.561 | 0.723 | 1.000 | 5946 | tags=13%, list=17%, signal=16% |
| 114 | KEGG\_ADHERENS\_JUNCTION |  | 68 | -0.22 | -0.86 | 0.594 | 0.779 | 1.000 | 9117 | tags=18%, list=26%, signal=24% |
| 115 | KEGG\_VEGF\_SIGNALING\_PATHWAY |  | 75 | -0.23 | -0.86 | 0.726 | 0.773 | 1.000 | 5587 | tags=16%, list=16%, signal=19% |
| 116 | KEGG\_GLIOMA |  | 65 | -0.21 | -0.85 | 0.685 | 0.773 | 1.000 | 4618 | tags=12%, list=13%, signal=14% |
| 117 | KEGG\_GLYCEROPHOSPHOLIPID\_METABOLISM |  | 70 | -0.21 | -0.84 | 0.712 | 0.797 | 1.000 | 4678 | tags=11%, list=13%, signal=13% |
| 118 | KEGG\_TYPE\_I\_DIABETES\_MELLITUS |  | 43 | -0.31 | -0.83 | 0.687 | 0.801 | 1.000 | 5214 | tags=23%, list=15%, signal=27% |
| 119 | KEGG\_NEUROTROPHIN\_SIGNALING\_PATHWAY |  | 125 | -0.17 | -0.83 | 0.658 | 0.808 | 1.000 | 8332 | tags=17%, list=24%, signal=22% |
| 120 | KEGG\_TYPE\_II\_DIABETES\_MELLITUS |  | 47 | -0.27 | -0.82 | 0.715 | 0.822 | 1.000 | 4395 | tags=15%, list=13%, signal=17% |
| 121 | KEGG\_FATTY\_ACID\_METABOLISM |  | 42 | -0.24 | -0.81 | 0.662 | 0.820 | 1.000 | 9857 | tags=31%, list=28%, signal=43% |
| 122 | KEGG\_AMYOTROPHIC\_LATERAL\_SCLEROSIS\_ALS |  | 53 | -0.23 | -0.81 | 0.746 | 0.820 | 1.000 | 8282 | tags=23%, list=24%, signal=30% |
| 123 | KEGG\_CITRATE\_CYCLE\_TCA\_CYCLE |  | 30 | -0.20 | -0.81 | 0.696 | 0.815 | 1.000 | 14056 | tags=47%, list=40%, signal=78% |
| 124 | KEGG\_LONG\_TERM\_POTENTIATION |  | 70 | -0.19 | -0.80 | 0.809 | 0.819 | 1.000 | 3581 | tags=10%, list=10%, signal=11% |
| 125 | KEGG\_TRYPTOPHAN\_METABOLISM |  | 39 | -0.24 | -0.80 | 0.824 | 0.824 | 1.000 | 8631 | tags=33%, list=25%, signal=44% |
| 126 | KEGG\_FOCAL\_ADHESION |  | 196 | -0.23 | -0.80 | 0.711 | 0.818 | 1.000 | 4618 | tags=12%, list=13%, signal=14% |
| 127 | KEGG\_RETINOL\_METABOLISM |  | 55 | -0.25 | -0.79 | 0.880 | 0.815 | 1.000 | 1602 | tags=11%, list=5%, signal=11% |
| 128 | KEGG\_DRUG\_METABOLISM\_CYTOCHROME\_P450 |  | 62 | -0.24 | -0.78 | 0.882 | 0.838 | 1.000 | 1602 | tags=8%, list=5%, signal=8% |
| 129 | KEGG\_PATHOGENIC\_ESCHERICHIA\_COLI\_INFECTION |  | 54 | -0.18 | -0.77 | 0.725 | 0.842 | 1.000 | 3726 | tags=7%, list=11%, signal=8% |
| 130 | KEGG\_SNARE\_INTERACTIONS\_IN\_VESICULAR\_TRANSPORT |  | 38 | -0.22 | -0.77 | 0.712 | 0.836 | 1.000 | 1960 | tags=5%, list=6%, signal=6% |
| 131 | KEGG\_TASTE\_TRANSDUCTION |  | 52 | -0.25 | -0.73 | 0.934 | 0.894 | 1.000 | 3581 | tags=19%, list=10%, signal=21% |
| 132 | KEGG\_SPLICEOSOME |  | 125 | -0.17 | -0.72 | 0.868 | 0.897 | 1.000 | 15376 | tags=47%, list=44%, signal=84% |
| 133 | KEGG\_ERBB\_SIGNALING\_PATHWAY |  | 86 | -0.16 | -0.71 | 0.897 | 0.905 | 1.000 | 4618 | tags=9%, list=13%, signal=11% |
| 134 | KEGG\_T\_CELL\_RECEPTOR\_SIGNALING\_PATHWAY |  | 107 | -0.19 | -0.70 | 0.922 | 0.917 | 1.000 | 6722 | tags=19%, list=19%, signal=23% |
| 135 | KEGG\_STARCH\_AND\_SUCROSE\_METABOLISM |  | 39 | -0.23 | -0.70 | 0.884 | 0.910 | 1.000 | 2738 | tags=10%, list=8%, signal=11% |
| 136 | KEGG\_JAK\_STAT\_SIGNALING\_PATHWAY |  | 151 | -0.21 | -0.69 | 0.936 | 0.911 | 1.000 | 8528 | tags=28%, list=25%, signal=37% |
| 137 | KEGG\_ABC\_TRANSPORTERS |  | 44 | -0.23 | -0.69 | 0.942 | 0.906 | 1.000 | 9368 | tags=34%, list=27%, signal=47% |
| 138 | KEGG\_PPAR\_SIGNALING\_PATHWAY |  | 69 | -0.21 | -0.67 | 0.965 | 0.923 | 1.000 | 7944 | tags=25%, list=23%, signal=32% |
| 139 | KEGG\_ECM\_RECEPTOR\_INTERACTION |  | 84 | -0.18 | -0.58 | 0.979 | 0.988 | 1.000 | 5798 | tags=17%, list=17%, signal=20% |
| 140 | KEGG\_AMINO\_SUGAR\_AND\_NUCLEOTIDE\_SUGAR\_METABOLISM |  | 42 | -0.16 | -0.55 | 0.933 | 0.993 | 1.000 | 16539 | tags=67%, list=48%, signal=127% |
| 141 | KEGG\_GLYCOSPHINGOLIPID\_BIOSYNTHESIS\_GANGLIO\_SERIES |  | 15 | -0.15 | -0.42 | 1.000 | 1.000 | 1.000 | 29418 | tags=100%, list=85%, signal=654% |
| 142 | KEGG\_BLADDER\_CANCER |  | 41 | -0.13 | -0.37 | 0.994 | 1.000 | 1.000 | 30150 | tags=100%, list=87%, signal=758% |
| 143 | KEGG\_RENAL\_CELL\_CARCINOMA |  | 66 | -0.11 | -0.35 | 0.998 | 1.000 | 1.000 | 14077 | tags=39%, list=41%, signal=66% |
Table: Gene sets enriched in phenotype **l (8 samples) **[plain text format]****
